# Ohio State Dental Society

**Published:** 1884-12

**Authors:** J. R. Callahan


### PROCEEDINGS.

### Ohio State Dental Society.

REORGANIZED.

A meeting of Dentists was held in Columbus, Ohio, Oct. 30, 1884, for the purpose of organizing a State Dental Society.

On motion of Dr. C. R. Butler, Dr. C. H. James was elected President; J. R. Callahan, Sec'y; Geo. W. Keely, Treas.

Dr. H. A. Smith moved that all members of the old Ohio State Dental Society present, become incorporators of the Ohio State Dental Society (reorganized). Motion prevailed.

On motion of Prof. J. Taft, a committee of three was appointed to draft Constitution and By-laws, consisting of Drs. H. A. Smith, Frank A. Hunter, F. H. Rehwinkle.

On motion of Dr. Williams, the President was added to the above committee.

At the request of the meeting, Dr. Emminger procured the services of a notary public to draw up articles of incorporation. Dr. Geo. Watt moved that a committee of three be appointed to assist the notary in preparing articles of incorporation. Carried.

The following committee was then appointed: Drs. J. Taft, W. H. Sillito, and H. H. Harrison.

On motion of Dr. C. R. Butler, it was decided that the first annual meeting of this Society be held in Chillicothe, Ohio, on the last Wednesday of October, 1885.

Prof. J. Taft then read the Articles of Incorporation, as prepared by the committe, as follows:

THE OHIO STATE DENTAL SOCIETY.

We, the undersigned, residents of the State of Ohio, do, by these presents, pursuant to and in conformity with the Acts of the Legislature of the State of Ohio authorizing the creation of corporations, and the several Acts of said Legislature amendatory thereof, associate ourselves together and form a body politic and corporate, and do hereby certify—

First.—That the name of said corporation shall be The Ohio State Dental Society.

Second.—The corporation is formed for the purpose of mutual fellowship, the promotion of the honor, usefulness, and interests of the profession :—Geo. Watt, C. H. James, C. H. Harroun, I. Williams, J. Taft, David Gibbons, A. F. Emminger, E. G. Betty, H. H. Harrison, B. R. Taber, W. H. Sillito, Frank A. Hunter, H. A. Smith, F. H. Rehwinkle, Geo. W. Keely, A. Berry. J. R. Callahan and C. R. Butler.

Dr. Frank A. Hunter moved that the officers elected at the last session of the old or defunct Ohio State Dental Society be elected to fill the same respective positions in the present or new society. Motion prevailed.

The President was instructed to appoint the usual committees.

Adjourned.

J. R. CALLAHAN, Secretary.

